# Paracheck-Pf^® ^accuracy and recently treated *Plasmodium falciparum *infections: is there a risk of over-diagnosis?

**DOI:** 10.1186/1475-2875-6-58

**Published:** 2007-05-16

**Authors:** Todd D Swarthout, Helen Counihan, Raphael Kabangwa K Senga, Ingrid van den Broek

**Affiliations:** 1 Médecins Sans Frontières, London, UK; 2 Epicentre, Paris, France; 3 AMI-KIVU Laboratories, Goma, Democratic Republic of Congo

## Abstract

**Background:**

An assessment of the accuracy of *Paracheck Pf*^®^, a malaria rapid diagnostic test (RDT) detecting histidine rich protein 2 was undertaken amongst children aged 6–59 months in eastern Democratic Republic of Congo.

**Methods:**

This RDT assessment occurred in conjunction with an ACT efficacy trial. Febrile children were simultaneously screened with both RDT and high quality microscopy and those meeting inclusion criteria were followed for 35 days.

**Results:**

358 febrile children were screened with 180 children recruited for five weeks follow-up. On screening, the RDT accurately diagnosed all 235 true malaria cases, indicating 100% RDT sensitivity. Of the 123 negative slides, the RDT gave 59 false-positive results, indicating 52.0% (64/123) RDT specificity. During follow-up after treatment with an artemisinin-based combination therapy, 98.2% (110/112), 94.6% (106/112), 92.0% (103/112) and 73.5% (50/68) of effectively treated children were still false-positive by RDT at days 14, 21, 28 and 35, respectively.

**Conclusion:**

Results show that though the use of *Paracheck-Pf*^® ^is as sensitive as microscopy in detecting true malaria cases, a low specificity did present a high frequency of false-positive RDT results. What's more, a duration of RDT false-positivity was found that significantly surpassed the 'fortnight' after effective treatment reported by its manufacturer. Though further research is needed in assessing RDT accuracy, study results showing the presence of frequent false positivity should be taken into consideration to avoid clinicians inappropriately focusing on malaria, not identifying the true cause of illness, and providing unnecessary treatment.

## Background

In response to increased antimalarial drug resistance, the past decade has witnessed the introduction of artemisinin-based combination therapy (ACT) by many malaria-endemic countries. With the significantly higher cost of ACT over the previously used chloroquine and sulphadoxine-pyrimethamine, there has been a strong emphasis in avoiding any unnecessary use of ACT and minimizing opportunities for the development of parasite drug resistance. Though clinical diagnosis can be very sensitive, its limited specificity can lead to inappropriate treatment [1]. As such, clinicians can no longer afford to provide treatment on the basis of clinical diagnosis alone. ACT implementation should go hand-in-hand with a greater capacity for biological confirmation of malaria diagnosis. Microscopy, still recognized as the gold standard, requires a functioning laboratory with ongoing support. Where this is not possible, an alternative is the use of a rapid diagnostic test which is simple to perform, requires no equipment or electricity and gives a result within 15 to 20 minutes.

Although there has been a marked increase in the number of RDTs commercially available, they can be roughly divided into two categories. One group of RDTs, including *Paracheck-Pf*^® ^(Orchid Biomedical Systems, Goa, India), detects the histidine rich protein 2 (HRP2), a protein uniquely synthesized by *Plasmodium falciparum *and present in the bloodstream of an infected individual [2,3]. Some HRP2 test kits are designed to also detect aldolase, a protein synthesized by all four human-infecting *Plasmodium *species. The second group of RDT detects parasite lactate dehydrogenase (pLDH), an enzyme produced by all four human malaria species.

Although HRP2 test kits have generally shown higher sensitivity for *P. falciparum *and can be less costly than the pLDH alternative, studies have shown that HRP2 remains in the bloodstream for an extended time following successful eradication of the parasite, contributing to false positive results and limited specificity. A study by Humar *et al. *(1997) on ParaSight F (early HRP2 version utilizing IgG antibodies) showed detectable levels of HRP2 in 27% of patients 28 days following successful treatment [4]. Though HRP2 kits currently use IgM antibodies which demonstrate higher specificity, subsequent studies still show similar duration of false positive results, with a day-14 and a day-21 false positivity of 35% and 16%, respectively [5,6]. While assessing *Paracheck-Pf *and two other RDTs, a 2002 study in Vietnam went further to report a strong correlation between extended duration of positivity and higher parasite density at presentation [7].

To date, no comprehensive study of persistent HRP2 antigenaemia has been published from sub-Saharan Africa. Therefore, coinciding with an ACT efficacy study, the accuracy of *Paracheck-Pf*^® ^was assessed, comparing RDT diagnosis to high quality microscopy during initial screening and follow-up.

## Methods

### Study site

This RDT assessment occurred parallel to a 28-day ACT efficacy trial that followed WHO guidelines [8]. The methodology and results of the ACT efficacy trial are described elsewhere [9]. In brief, the Democratic Republic of Congo (DRC) is Africa's third largest country with an estimated 2002 population of 53 million. Malaria is high-endemic and seasonal throughout the DRC, with peaks during the low (March to May) and high (September to November) rainy season. A prolonged civil war has lead to large numbers of internally displaced persons and degradation of infrastructure, including the health delivery systems. The study site was the small town of Shabunda in South Kivu Province, a very isolated community accessible only by plane or on foot. *Plasmodium falciparum *accounts for 95% of the plasmodium species in this region. Study approval was obtained from the ethics committee of the DRC National Malaria Programme and the external Ethics Review Board used by Médecins Sans Frontières.

### Study design

The RDT assessment was performed from March to June 2004. After screening of febrile children six to 59 months of age, 180 children who met inclusion criteria for the ACT efficacy study (including uncomplicated clinical *P. falciparum *malaria confirmed by microscopy, unmixed *P. falciparum *infection between 2,000 – 200,000 parasites per μL of blood, no concomitant disease that could mask the response to antimalarial treatment) were recruited after their guardians provided written informed consent. Children were randomized to receive artesunate + amodiaquine or artesunate + sulphadoxine-pyrimethamine. Children were followed to assess both ACT efficacy and duration of false positive RDT results, as defined by positive RDT and continued negative microscopy after ACT treatment. For the purposes of the RDT assessment, follow-up was performed by study clinicians on days 7, 14, 21, 28 post-treatment or on any other day if the child was unwell. An additional visit at day-35 was added specifically to continue monitoring RDT results.

### Laboratory methods

At all sampling points, a blood smear for microscopic detection of parasites was made and stained with 10% pre-filtered Giemsa. Both asexual parasites and gametocytes were counted against 200–500 leucocytes and converted to number of parasites per volume assuming 8,000 leucocytes/μL blood. Slides were considered negative when no parasites were detected after viewing 100 microscopic fields. Microscopists unaware of treatment allocation read all slides. Internal quality control included a blind second reading of a proportion of the slides: all slides taken during day 0 and day 3; all positive slides after day 3; 20% of negative slides. All day 28 and day 35 negative slides were reread. In cases where there was discordance, the two technicians re-examined the slides together and made a collective decision on the reading. Discordance included any discrepancies in final result (i.e. negative vs. positive), difference in malaria species, or a difference in parasite density greater than 25%. External controls were conducted by a Ministry of Health government reference laboratory in the provincial capital.

Concurrent with blood smear collection, RDTs were completed with the same finger prick blood sample at time of screening and follow-up. Test kits were stored as directed by the manufacturer and quality of package desiccant was checked before use. The fresh blood sample was transferred directly to the sample pad by the provided sample applicator. All RDTs were labelled with patient ID numbers and results were recorded 15 minutes after adding 6 drops (300 μL) of clearing buffer. Presence of both the control and test lines indicated a positive result for *P. falciparum *and presence of only the control line indicated a negative result. In any case where the control line did not appear, the result was considered invalid and the test was repeated. The person recording the RDT result was unaware of corresponding microscopy results. Internal quality control included an immediate blind second reading of 100% of RDTs. In cases where there was discordance, the two technicians re-examined the RDT together and made a collective decision on the reading.

### Data analysis

Data was double-entered into Excel (Microsoft XP) and transferred to STATA (version 8.0, STATA Corporation, College Station, Texas, USA) for further analyses. Data included in analysis for duration of false positive RDT results is only from children who remained slide-negative until at least 28 days after treatment.

## Results

Between March and April 2004, 358 febrile children were screened for *P. falciparum *infection with both RDT and microscopy (Table 1). Nine RDTs gave indeterminate results (three showed no control line and six were unclear because the blood did not clear the field) and were successfully repeated. Of the 358 children screened, 235 were *P. falciparum *positive by microscopy. As shown in Table 2, the RDT accurately diagnosed all 235 true malaria cases, indicating an RDT sensitivity of 100% (235/235; 95% CI 98.4–100). Of the 123 negative slides, 64 were negative by RDT, giving 59 false-positive RDT results and an RDT specificity of 52.0% (64/123; 95% CI 42.8–61.1).

**Table 1:** RDT and microscopy results at screening (N = 358)

	Slide positive, P.f.	Slide negative, P.f.	Total
RDT Positive	235	59	294
RDT Negative	0	64	64

Total	235	123	358

**Table 2:** RDT Accuracy at Screening (N = 358)

Parameter		Index	
Sensitivity	......................................................................	100	(98.4–100)
Specificity	......................................................................	52.0	(42.8–61.1)
Negative predictive value	.................................................................	100	(94.4–100)
Positive predictive value	.................................................................	79.9	(74.9–84.4)

At screening, the positive predictive value (PPV) of the RDT was 79.9% (235/294; 95% CI 74.9–84.4), i.e. 79.9% of the 294 positive RDT results were matched by positive microscopy results (Table 2). All 64 negative RDT results were matched by negative microscopy results, indicating a negative predictive value (NPV) of 100% (64/64; 95% CI 94.4–100).

180 children were recruited for a five-week follow-up. 68 children were removed from final analysis for the following reasons: 42 children were retreated within 28 days due to malaria recrudescence or reinfection, later confirmed by PCR; five were withdrawn from the study (two for vomiting the treatment dose twice at enrolment, one due to incorrect inclusion into the study, two for intake of non-study antimalarials during follow-up); one child was lost to follow-up due to family movement; 20 children were not able to complete a day-28 clinic visit after forced evacuation of the study team due to political insecurity in the region. This evacuation prevented an additional 44 children from having a day-35 visit. In summary, for day-28 and day-35 clinic visits, 112 and 68 children attended, respectively.

**Figure 1:**
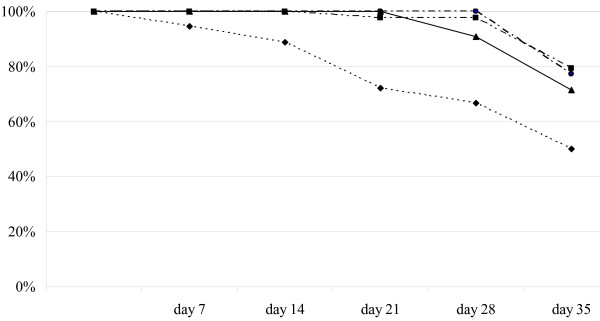
Proportion of children with continued false-positive RDTs at clinic visit, stratified by day-0 parasite density. Legend of parasite density: ● 50–20000; ■ 20–50000; ▲ 10–20000; ◆ 2–10000

At days 7, 14, 21, 28, and 35 after effective treatment, 99.1% (111/112; 95% CI 95.1–100), 98.2% (110/112; 95% CI 93.7–-99.8), 94.6% (106/112; 95% CI 88.7–98.0), 92.0% (103/112; 95% CI 85.3–96.3), and 73.5% (50/68; 95% CI 61.4–83.5) of the patients attending a clinical visit were still false-positive by RDT, respectively (Table 3, bottom row). Figure 1 and Table 3 show the duration and proportion of false-positive results during follow-up as a function of parasite density at screening. Day-0 parasite density (per μL) is stratified: 2,000–10,000 (n = 18 children); 10,000–20,000 (n = 22); 20,000–50,000 (n = 42) and 50,000–200,000 (n = 30). 28 days after effective treatment, 80.0% (32/40; 95% CI 64.4–90.9) of the children with lower parasite density had false positive RDT results, whereas 98.6% (71/72; 95% CI 92.5–99.9) of those with higher parasite density continued to show false positive results (Table 3). This represents a significant difference (p = 0.002).

**Table 3:** Proportion of children with continued false-positive (FP) RDTs, stratified by day-0 parasite density

Parasite density (× 10^3^), Day 0	Day 7 % FP (95% CI)	Day 14 % FP (95% CI)	Day 21 % FP (95% CI)	Day 28 % FP (95% CI)	Day 35 % FP* (95% CI)
50–200 (n = 30)	100	100	100	100	77.3 (17/22)
	-	-	-	-	(54.6–92.2)
20–50 (n = 42)	100	100	97.6 (41/42)	97.6 (41/42)	79.2 (19/24)
	-	-	(87.4–99.9)	(87.4–99.9)	(57.8–92.9)
10–20 (n = 22)	100	100	100	90.9 (20/22)	71.4 (10/14)
	-	-	-	(70.9–98.9)	(46.2–95.0)
2–10 (n = 18)	94.4 (17/18)	88.9 (16/18)	72.2 (13/18)	66.7 (12/18)	50.0 (4/8)
	(72.7–99.9)	(65.3–98.6)	(46.5–90.3)	(41.0–86.7)	(15.7–84.3)
Total	99.1 (111/112)	98.2 (110/112)	94.6 (106/112)	92.0 (103/112)	73.5 (50/68)
	(95.1–100)	(93.7–99.8)	(88.7–98.0)	(85.3–96.3)	(61.4–83.5)

*44 children were not able to complete a day-28 clinic visit after forced evacuation of the study team due to political insecurity in the region.

There was no significant correlation between presence of gametocytes or schizonts and duration of RDT positivity.

## Discussion

Results from this study show that the use of Paracheck-*Pf*^® ^is as sensitive as microscopy in detecting true falciparum malaria cases in this population. This is paramount, as no true malaria cases would go untreated in this vulnerable population of children under 5 years of age. However, the frequent occurrence of false positive results can lead to a number of true non-malaria cases being treated unnecessarily. This can have several negative outcomes, including clinicians inappropriately focusing on malaria and not identifying the true cause of illness and unnecessary exposure to antimalarials. In some cases, the inappropriately treated patient may return with similar symptoms, leading the clinician to falsely report the presence of parasite drug resistance. This could lead to the clinician not trusting the efficacy of the first-line antimalarial and consequently dispensing the second-line antimalarial, increasing the cost of treatment and further delaying appropriate treatment.

While other studies have shown relatively high RDT sensitivity [7], this study showed 100% RDT sensitivity when compared to microscopy at time of screening. However, results from this study also show a relatively low 52.0% specificity. One explanation for this low specificity might include prior infection with effective treatment. That is to say, though exclusion criteria included reported malaria treatment within the previous 14 days, it is possible that some children were infected and adequately treated between 14 and 35 days prior to screening for inclusion. As study results show, the majority of these children would still have false positive RDT results.

Using PCR, Bell *et al. *(2005) showed that false positive results can be explained by the presence of blood samples with parasite density levels below the detection threshold for microscopy [10]. Such subpatent levels of parasites could still produce detectable levels of HRP2. However, this work was done in a community with fluctuating low parasite density. Shabunda is endemic and anecdotal clinical evidence suggests that there are very few children less than 5 years of age that carry stable subpatent malaria infections. While it is possible that some children at screening had malaria infections that were subpatent and asymptomatic, yet developing into clinical malaria, such instances are likely to be few and would not significantly change the prevalence of false positive RDTs.

These study results show that the duration of Paracheck false positivity can be more than 35 days after effective treatment. As mentioned in the introduction, past evidence strongly suggests that this is due, in large part, to the prolonged time it takes for HRP2 to be cleared from the blood following treatment of falciparum malaria. Though the mechanism of HRP2 clearance is not well understood, there are several feasible explanations for its long persistence after adequate therapy, as discussed below.

Duration of RDT false positivity has previously been correlated to higher parasite density on admission. As secretion of the protein is proportional to parasite numbers [11], a higher parasite density on admission would cause an extended period of time needed for HRP2 clearance from the blood. Results from this study further support this, showing a strong correlation between duration of Paracheck positivity and parasite density at admission. As most previous studies have been done outside sub-Saharan Africa where the parasite burdens are much lower, this may contribute to explaining the surprisingly high false positivity rate found in this study,

HRP2 is also released by early gametocytes [12] and development from young to mature gametocytes for *P. falciparum *requires a longer time (about 8–10 days) than for other malaria species [13]. Thus persistence of immature gametocytes in the blood after successful therapy could result in persistence of HRP2 positivity. However, this study showed no association between gametocytaemia and duration of RDT positivity.

Earlier reports suggested that persistence of HRP2 may result from a cross-reacting auto-antibody such as rheumatoid factor (RF) present in the blood [14]. However, the antibodies used in Paracheck are pre-absorbed against RF antigens which would neutralize such cross-reactivity. Further research would be needed to assess the presence of other auto-antibodies within the study population that may cause a similar cross reactivity with this type of RDT.

Persistent antigenaemia could result from slow or incomplete clearance of circulating or sequestered sensitive parasites. After treatment, some patients with relatively resistant parasites might have persistent sub-patent parasite density (<100 parasites/μl) and a recrudescence of their infection after day 28 or 35. However, late recrudescence is unlikely to have happened in such a large proportion of cases as the artemisinin-based drugs currently display near 100% efficacy. Singh and Shukla [5] found a shorter time of HRP2 test positivity after treatment with the rapid-acting artemisinin-compound artemether than after treatment with CQ or SP [15].

A duration of Paracheck-Pf false-positivity was found that significantly surpassed the 'fortnight' suggested by its manufacturer. Though other studies have shown the same trend, none have shown such a high proportion of RDTs still positive 28–35 days after parasite clearance. While further studies are needed, these results reinforce the need for medical staff to incorporate a patient's clinical history when interpreting Paracheck results. This is especially true for patients with routine clinical visits that include malaria screening and in areas with high malaria endemicity.

## Authors' contributions

**TDS **Responsible for study design, field-implementation, supervision of the team in the field, data collection, analyses, writing of and finalising this paper. **HC **Responsible for supporting data analysis and writing of finalised paper. **RKKS **Contributed to laboratory protocol design and supported supervision of laboratory team. **IvdB **Contributed to study design, overall supervision of the study, and supported data analysis and writing of this paper. All authors read and approved the final manuscript.
